# Maternal Disease With Group B *Streptococcus* and Serotype Distribution Worldwide: Systematic Review and Meta-analyses

**DOI:** 10.1093/cid/cix660

**Published:** 2017-11-06

**Authors:** Jennifer Hall, Nadine Hack Adams, Linda Bartlett, Anna C Seale, Theresa Lamagni, Fiorella Bianchi-Jassir, Joy E Lawn, Carol J Baker, Clare Cutland, Paul T Heath, Margaret Ip, Kirsty Le Doare, Shabir A Madhi, Craig E Rubens, Samir K Saha, Stephanie Schrag, Ajoke Sobanjo-ter Meulen, Johan Vekemans, Michael G Gravett

**Affiliations:** 1 Department of Reproductive Health Research, University College London Institute for Women’s Health, United Kingdom;; 2 School of Social and Community Medicine, University of Bristol, United Kingdom;; 3 Department of International Health, Johns Hopkins Bloomberg School of Public Health, Baltimore, Maryland;; 4 Maternal, Adolescent, Reproductive and Child Health Centre, London School of Hygiene & Tropical Medicine, United Kingdom;; 5 College of Health and Medical Sciences, Haramaya University, Dire Dawa, Ethiopia;; 6 Healthcare-Associated Infection and Antimicrobial Resistance Department, National Infection Service, Public Health England, London,United Kingdom;; 7 Departments of Pediatrics and Molecular Virology and Microbiology, Baylor College of Medicine, Houston, Texas;; 8 Medical Research Council: Respiratory and Meningeal Pathogens Research Unit, and Department of Science and Technology/National Research Foundation: Vaccine Preventable Diseases, Faculty of Health Sciences, University of the Witwatersrand, Johannesburg, South Africa;; 9 Vaccine Institute, Institute for Infection and Immunity, St George’s Hospital, University of London and St George’s University Hospitals NHS Foundation Trust, United Kingdom;; 10 Department of Microbiology, Faculty of Medicine, Chinese University of Hong Kong;; 11 Centre for International Child Health, Imperial College London, United Kingdom;; 12 National Institute for Communicable Diseases, National Health Laboratory Service, Johannesburg, South Africa;; 13 Global Alliance to Prevent Prematurity and Stillbirth, Seattle, Washington;; 14 Department of Global Health, University of Washington, Seattle;; 15 Bangladesh Institute of Child Health, Dhaka;; 16 National Center for Immunization and Respiratory Diseases, Centers for Disease Control and Prevention, Atlanta, Georgia;; 17 Bill & Melinda Gates Foundation, Seattle, Washington;; 18 World Health Organization, Geneva, Switzerland; and; 19 Department of Obstetrics and Gynecology, University of Washington, Seattle

**Keywords:** group B *Streptococcus*, pregnancy, postpartum, incidence, serotype

## Abstract

**Background:**

Infections such as group B *Streptococcus* (GBS) are an important cause of maternal sepsis, yet limited data on epidemiology exist. This article, the third of 11, estimates the incidence of maternal GBS disease worldwide.

**Methods:**

We conducted systematic literature reviews (PubMed/Medline, Embase, Latin American and Caribbean Health Sciences Literature [LILACS], World Health Organization Library Information System [WHOLIS], and Scopus) and sought unpublished data on invasive GBS disease in women pregnant or within 42 days postpartum. We undertook meta-analyses to derive pooled estimates of the incidence of maternal GBS disease. We examined maternal and perinatal outcomes and GBS serotypes.

**Results:**

Fifteen studies and 1 unpublished dataset were identified, all from United Nations–defined developed regions. From a single study with pregnancies as the denominator, the incidence of maternal GBS disease was 0.38 (95% confidence interval [CI], .28–.48) per 1000 pregnancies. From 3 studies reporting cases by the number of maternities (pregnancies resulting in live/still birth), the incidence was 0.23 (95% CI, .09–.37). Five studies reported serotypes, with Ia being the most common (31%). Most maternal GBS disease was detected at or after delivery.

**Conclusions:**

Incidence data on maternal GBS disease in developing regions are lacking. In developed regions the incidence is low, as are the sequelae for the mother, but the risk to the fetus and newborn is substantial. The timing of GBS disease suggests that a maternal vaccine given in the late second or early third trimester of pregnancy would prevent most maternal cases.

## Teaser

From the limited data available, the incidence of maternal group B streptococcal (GBS) disease in developed countries appears lower than, but comparable to, the incidence of early-onset neonatal GBS disease. There are no published data from low- and middle-income contexts.

Maternal sepsis is an important and potentially preventable cause of global maternal mortality. Although data are limited, particularly from countries with the highest maternal mortality ratios, maternal sepsis was estimated to cause around 11% (95% confidence interval [CI], 6%–19%, n = 261000) of maternal deaths worldwide between 2003 and 2009 [1]. It is especially prevalent in South Asia where it accounts for 14% of all maternal deaths (95% CI, 3%–36%) and sub-Saharan Africa (10% [95% CI, 5.5%–18.5]) [1]. In comparison, the proportion of maternal deaths due to sepsis in developed countries was estimated at 4.7% (95% CI, 2.4%–11.1%) [1].

In northwestern Europe, >40% of all maternal deaths were caused by puerperal sepsis in the early 1900s [2]. Serial data from England and Wales, one of the few areas to have extensive historical data on maternal mortality, show that puerperal sepsis caused 55% of deaths in the 1870s but only 4.6% by the 1980s [3]. This decline is attributed primarily to knowledge of hygienic childbirth practice and antibiotics. However, sepsis has reemerged as a leading cause of maternal death in the United Kingdom, accounting for nearly 25% of deaths in 2009–2012 [4] and is now the second most common cause of death [5]. This may be due to a number of factors, including (1) changes in maternal risk factors for sepsis, such as age at first pregnancy, the prevalence of comorbidities including obesity and diabetes, the ethnic makeup of a population, and levels of multiple births; (2) alterations in the virulence of circulating organisms; and/or (3) variation in iatrogenic factors such as the use of repeated invasive diagnostic and therapeutic procedures. A corresponding increase in the incidence and severity of sepsis in the general population has been noted in Europe and in the United States [6–8].

In keeping with the general decline in maternal deaths due to sepsis in developed countries over the last 150 years, the incidence of maternal sepsis has fallen from 0.8% in the 1970s [9, 10] to 0.1%–0.3% [11–14] in the 2000s. However, up to 10% of all pregnant women are reported to experience febrile morbidity [15], representing a significant burden of ill health. Beyond maternal mortality, maternal infection can have short and long-term effects not only on maternal health but also on the outcome of the pregnancy (eg, preterm labor, stillbirth, neonatal sepsis) and the longer-term health and development of the child [16–19].

Despite the burden of maternal, perinatal, and neonatal mortality and morbidity associated with maternal sepsis, data on the etiology, particularly in low- and middle-income contexts, are limited [20]. Group B *Streptococcus* (GBS; *Streptococcus agalactiae*), part of the normal flora in the intestine, vagina, and rectum, is likely an important pathogen in maternal sepsis because around 1 in 5 pregnant women are colonized worldwide [21], and in pregnancy there is increased risk of invasive GBS disease [11, 22–25]. Indeed, GBS is frequently identified as a pathogen in maternal sepsis [9, 10, 14, 19, 26]; GBS accounted for 25% of clinically significant bacteremia in hospitalized pregnant women in Ireland [27] and 20% of hospitalized women with puerperal bacteremia in the United States [19]. Few publications, however, have specifically estimated the incidence of maternal GBS disease.

**Figure 1.**
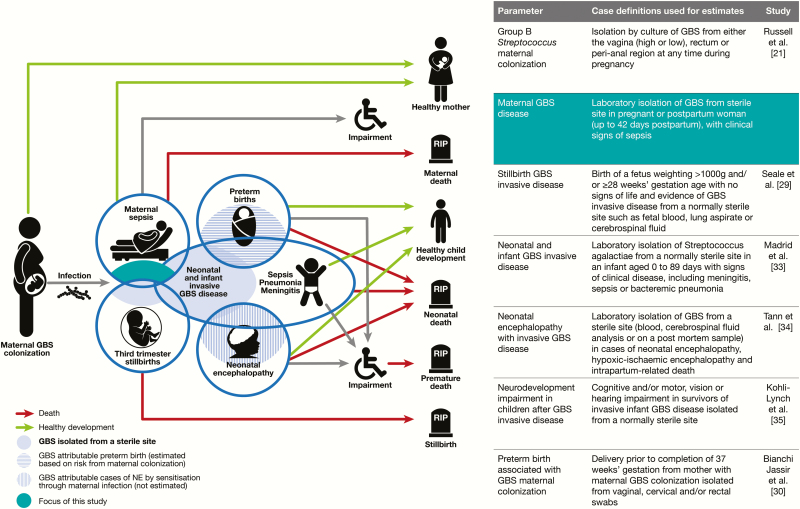
Maternal group B streptococcal (GBS) disease in disease schema for GBS, as described by Lawn et al [28]. Abbreviations: GBS, group B *Streptococcus*; NE, neonatal encephalopathy.

This article, assessing the incidence of invasive maternal GBS disease worldwide (Figure 1), is part of a supplement estimating the burden of GBS disease in pregnant and postpartum women, stillbirths, and infants, which is important in terms of public health policy and particularly vaccine development [28]. The supplement includes systematic reviews and meta-analyses on GBS colonization and adverse outcomes associated with GBS around birth [21, 29–35], which form input parameters to a compartmental model [36]. These are reported individually and according to international guidelines [37, 38].

The specific objectives of this article are:

1. To provide a comprehensive and systematic literature review and meta-analyses to assess the incidence of maternal GBS disease per 1000 pregnancies, the associated maternal, perinatal, and neonatal outcomes and the serotype distribution of maternal GBS disease;
2. To use the data input available for estimating the burden of GBS in pregnancy and postpartum for women, stillbirth, and infants; and
3. To evaluate the gaps in the data and recommend what should be done to improve the data on maternal GBS disease.

## METHODS

This article is part of a wider study protocol entitled “Systematic estimates of the burden of GBS worldwide in pregnant and postpartum women, stillbirths and infants.” It was submitted for ethical approval to the London School of Hygiene & Tropical Medicine (reference number 11966) and approved on 30 November 2016.

### Definitions

Maternal GBS disease was defined as laboratory isolation of GBS from a sterile site (blood or cerebrospinal fluid [CSF] only) in a pregnant or postpartum woman (up to 42 days postpartum), with a minimum of fever and physician suspicion of sepsis. Nonsystemic infections, such as chorioamnionitis, pyelonephritis, or soft tissue infections, were excluded.

### Search Strategy

We identified data for this supplement through systematic review of the published literature and through development of an investigator group asking clinicians, researchers, and relevant professional institutions worldwide. For this article, systematic searches of Medline, Embase, the World Health Organization Library Information System (WHOLIS), Literature in the Health Sciences in Latin America and the Caribbean (LILACS), and Scopus were completed in November 2016, and updated to include all studies published to the end of January 2017. Search terms related to “pregnancy,” “maternal,” “peripartum,” “GBS,” and “sepsis” were used and medical subject headings (MeSH) terms were used where possible (see Supplementary Table 1 for the full search terms). Each article was reviewed and had data extracted by at least 2 reviewers. Where there was discrepancy between 2 reviewers, a third was consulted. The reference lists of relevant articles were hand-searched to identify additional studies.

### Inclusion and Exclusion Criteria

Any observational studies reporting the incidence of invasive GBS disease in pregnant women or women up to 42 days postpartum were eligible for inclusion. Reviews, case reports or series, and commentaries were excluded. No date or language restrictions were applied; texts were translated to English when published in other languages.

### Data Abstraction and Meta-analyses

Data from each study were extracted into standard Excel forms and imported to Stata 13 software (StataCorp) for meta-analyses. Where available, data were extracted and used to describe the maternal, perinatal, and neonatal outcomes for women with maternal GBS disease. Information on the serotype of GBS was extracted where reported.

We used random-effects meta-analyses to estimate the incidence of maternal GBS disease using the DerSimonian and Laird method [39]. The same approach was used to estimate the timing of disease in relation to the course of pregnancy (antepartum, peripartum, or postpartum), case fatality risks for maternal and neonatal mortality in maternal GBS disease, the incidence of early-onset GBS disease (EOGBS) in neonates born to women with maternal GBS disease, and the prevalence of GBS serotypes causing maternal GBS disease.

## RESULTS

**Figure 2.**
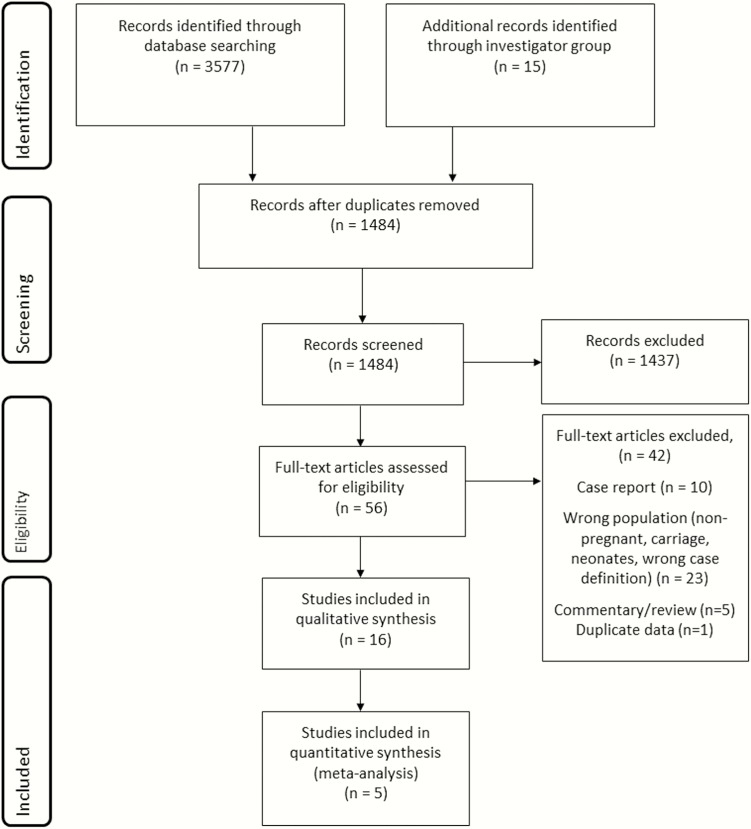
Data search and included studies for maternal group B streptococcal disease.

The database searches returned a total of 3580 hits combined; an additional 14 articles were identified through hand-searching the references, and 1 unpublished dataset was included. After duplicates were removed, 1488 papers remained. Following review of the title and abstract, the full text of 56 articles was reviewed. From these, 15 were retained, although only 4 were included in the meta-analysis of the incidence of invasive maternal GBS disease. In addition, one unpublished dataset from the United Kingdom [40] was included, for a total of 16 studies, 5 of which were included in the meta-analysis, as shown in Figure 2.

### Characteristics of Included Studies

**Figure 3.**
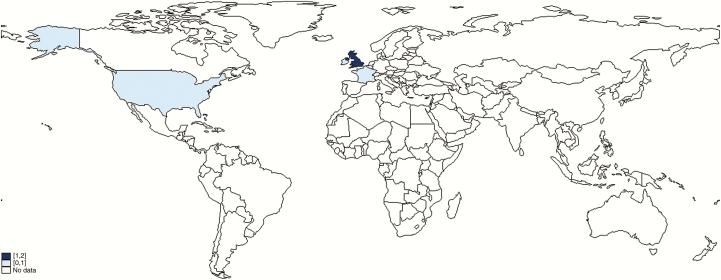
Geographic distribution of data on maternal group B streptococcal (GBS) disease that met inclusion criteria.

Thirteen studies from the systematic review met our inclusion criteria; an additional 2 provided relevant information on maternal GBS disease but no incidence estimate, plus one unpublished dataset from the United Kingdom [40] for a total of 16 studies, which are summarized in Table 1 [12, 14, 19, 22, 23, 25–27, 40–47]. The study periods ranged from 1981 to 2016; only 4 were published pre-2000 [19, 41, 44, 46]. All studies were hospital-based with many studies using the methodology of an audit of blood cultures from obstetric patients linked with a review of their medical records, some prospectively and some retrospectively. Different population denominators were used to estimate incidence: pregnancies, maternities (defined as women delivering either live or stillbirths), live births, total births, or per 1000 woman-years, and several studies only reported risks without providing the data that went into the estimate. We intended to estimate incidence rates per 1000 pregnancies; however only 1 study reported these data [26]. Four more used maternities as the denominator [12, 14, 40, 42], which we used for the meta-analysis. The number of maternities will be lower than the number of pregnancies, as pregnancies include miscarriages and induced abortions. The studies included in the meta-analysis of the incidence of maternal GBS disease were all published since 2013 (and included 1 set of unpublished data) and were conducted in the United States [12], France [14], Ireland [26], and the United Kingdom [40, 42] (Figure 3). Four were conducted retrospectively and 1 prospectively, and they were all large studies covering tens or hundreds of thousands of women.

**Table 1.** Characteristics of 16 Included Studies

First Author and Year	Year of Data Collection	Study Location	Study Design	No. of Pregnancies	No. of Live Births	No. of Live and Stillbirths	Inclusion Criteria	Cases of Maternal GBS Disease	Blood Cultures	Diagnosis of Maternal GBS Disease
Lamagni 2016 [40]	2014	All NHS patients in England	National population-based laboratory surveillance linked to hospital admission statistics	638863 deliveries	646455	649485	All women receiving NHS care in England	185	185	Laboratory- confirmed invasive GBS infection as determined through culture of GBS from normally sterile sites
Drew 2015 [27]	January 2001– December 2014	Rotunda Hospital, Dublin, Ireland	Retrospective audit of blood cultures taken from obstetric patients	Not reported	112361	Not reported	All clinically significant blood cultures taken from obstetric patients at Rotunda Hospital in the study period	64	64	Isolation of GBS from blood of an obstetric patient during the period of the audit
Kalin 2015 [42]	June 2011– May 2012	All UK consultant-led maternity units	Secondary analysis of GBS sepsis cases from population-based study (UKOSS facilitated). Case-control study	799003 maternities	Not reported	Not reported	All mothers delivering in UK hospitals	7	7	Severe sepsis and GBS isolated from sterile site in unwell mother
Knowles 2014 [26]	1 January 2005–31 December 2012	Coombe Women and Infants University Hospital and National Maternity Hospital, Dublin	Prospective review of medical records and laboratory data. Case- control study	136897 pregnancies	139495	139495	All mothers delivering in CWIUH and NMH	57 (2 antenatal; 43 intrapartum; 12 postpartum)	57	Laboratory- confirmed secondary bloodstream infection
O’Higgins 2014 [43]	1 January 2009–31 December 2012	Coombe Women and Infants University Hospital, Dublin, Ireland	Retrospective audit of blood cultures taken from obstetric patients	37584 pregnancies – note overlap with Knowles 2014 so excluded from meta-analysis	Not reported	Not reported	Blood cultures taken from obstetric patients which yielded a pathogenic organism and whose medical records were available for review	15 (10 intrapartum; 5 postpartum)	15	Isolation of GBS from the blood of an obstetric patient during the period of the audit
Cape 2013 [12]	January 2000– December 2008	Brigham and Women’s Hospital, Boston, Massachusetts	Retrospective cohort study	78781 maternities	81376	Not reported	Blood cultures taken from obstetric patients at the hospital in the study period; pathogenic organisms only	8	8	Isolation of GBS from blood of an obstetric patient during hospitalization
Surgers 2013 [14]	January 2005– December 2009	Five teaching hospitals across Paris	Retrospective multicenter audit of positive blood cultures and associated medical records of obstetric patients	59491 maternities	Not reported	Not reported	Blood cultures taken from obstetric patients which yielded a pathogenic organism and whose medical records were available for review	19 (17 intrapartum; 2 postpartum)	19	Isolation of GBS from the blood of an obstetric patient during the period of the audit
Deutscher 2011 [25]	2007–2009 (exact dates not specified)	California, Colorado, Connecticut, Georgia, Maryland, Minnesota, New Mexico, New York, Oregon, Tennessee	Multicenter, prospective, active surveillance study	Not reported	470646 (in 2007)	Not reported	Pregnant and postpartum women aged 15–44 y in surveillance areas with positive blood cultures. No mention of clinical criteria	99 (42 pre-/ intrapartum; 57 postpartum)	99	Isolation of GBS from a sterile site in a surveillance area resident (amniotic fluid and placenta not included)
Phares 2008 [22]	January 1999– December 2005	California, Colorado, Connecticut, Georgia, Maryland, Minnesota, New Mexico, New York, Oregon, Tennessee	Multicenter, prospective, active surveillance study	Not reported	454476	Not reported		409	211	Isolation of GBS from sterile site in a surveillance-area resident
Schrag 2000 [45]	1993–1998 (exact dates not specified)	Microbiology laboratories serving acute care hospitals in Maryland, Georgia, California, and Tennessee	Multicenter, prospective, active surveillance study	Not reported	Not reported	Not reported	All residents within surveillance areas of any age with GBS isolated from a sterile site (not including placenta or amniotic fluid)	345	221	Isolation of GBS from a sterile site in a surveillance area resident (amniotic fluid, placenta, and urine excluded)
Tyrrell 2000 [23]	1 January 1996–30 December 1996	Nine Canadian public health units	Multicenter, prospective, active surveillance study	Not reported	Not reported	Incidence rate of 41/100000 total births	All residents of surveillance area of any age, with GBS isolated from a sterile site. No mention of clinical criteria	15	11	Isolation of GBS from a sterile site in a surveillance area resident
Zaleznik 2000 [26]	January 1993– December 1996	12 hospitals in 4 cities in US (Houston, Minneapolis, Seattle, Pittsburgh)	Multicenter, prospective, active surveillance study	Not reported	157184	Incidence rate 0.3/1000 deliveries	All mothers delivering at the 4 included hospitals. Cases identified from microbiology laboratory records, febrile women (only 1 of 4 criteria for sepsis)	54	52	Isolation of GBS from blood or another usually sterile site (except urine) during hospitalization
Schwartz 1991 [46]	1982–1983	Atlanta, Georgia metropolitan area: all 37 acute care hospitals and independent bacteriology laboratories	Population- based surveillance for invasive GBS disease in adults	Not reported	Incidence of 22 cases/100000 live births	Not reported	Resident of the Atlanta health district from who GBS was isolated from a normally sterile site in 1982 or 1983	14	9	Isolation of GBS from a sterile site in a surveillance-area resident
Gallagher 1985 [41]	Jan 1980–June 1984	St Elizabeth Hospital Medical Center, a teaching hospital of northeastern Ohio	Retrospective audit of GBS-positive blood cultures	Not reported	Not reported	Not reported	Any person from whom GBS was isolated from blood culture specimens between January 1980 and June 1984 at St Elizabeth Hospital Medical Center	4	4	Isolation of GBS from the blood of any patient during time of audit
Pass 1982 [44]	June 1977– December 1979 and June 1977– June 1980	Cooper Green Hospital and University Hospital, University of Alabama, in Birmingham	Retrospective audit of patients with proven GBS sepsis, also results from prospective study of GBS infections	Not reported	Not reported	Incidence rates of 2.3 and 1.4/1000 deliveries, respectively for University Hospital and Cooper Green Hospital	Patients with proven GBS sepsis (also results for all nonbacteremic GBS infections in obstetric patients)	21	21	Proven GBS sepsis (not further defined, but all had GBS isolates from blood)
Gibbs 1981 [19]	March 1975– June 1979	Bexar County Teaching Hospitals, San Antonio, Texas	Retrospective audit of aerobic streptococcal infections in obstetric patients	Not reported	Not reported	Not reported	Patients with streptococcal infections with bacteremia in the hospital’s blood culture results system	31	31	Streptococcal isolate in 1 or more blood cultures accompanied by clinical signs of infection

Abbreviations: CWIUH, Coombe Women and Infants University Hospital; GBS, group B *Streptococcus*; NHS, National Health Service; NMH, National Maternity Hospital, Dublin; UK, United Kingdom; UKOSS, UK Obstetric Surveillance System; US, United States.

All 16 studies were used to provide data on other aspects and outcomes of maternal GBS disease. Five studies reported information on the timing of the disease with relation to the antenatal, delivery, or postnatal period [14, 26, 43, 44, 46]. Eleven articles reported the absolute number of maternal deaths [14, 22, 23, 25, 26, 40–44, 47], 6 articles reported some data on maternal morbidity. Nine articles provided data on perinatal outcomes from pregnant women with GBS disease [19, 23, 26, 40–42, 44, 46, 47]. Seven articles reported on neonatal mortality associated with maternal GBS disease [19, 23, 25, 41, 42, 44, 46], and 6 reported on cases of EOGBS disease in neonates born to pregnant women with GBS disease [19, 23, 41, 42, 44, 47]. Two articles reported on colonization [14, 44]. Serological typing of GBS bacterial isolates was undertaken in 5 studies [22, 23, 25, 44, 47].

### Incidence

**Figure 4.**
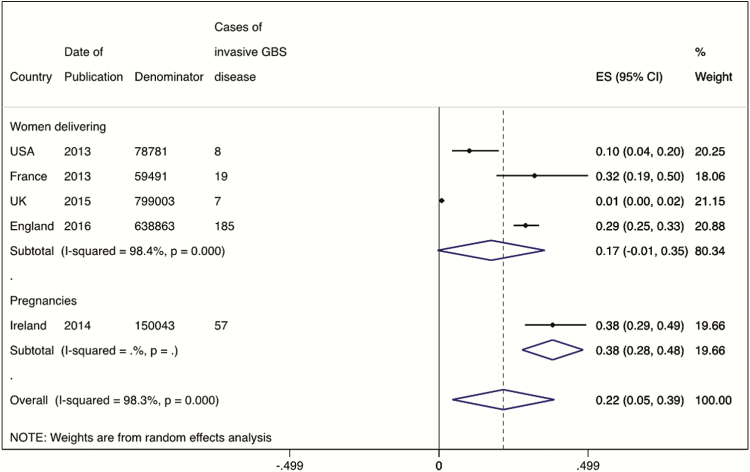
Meta-analysis of the incidence of maternal group B streptococcal disease, split by denominator of women delivering (4 studies, N = 1576138) or total pregnancies (1 study, n = 150043). Abbreviations: CI, confidence interval; ES, effect size; GBS, group B *Streptococcus*.

From the single study using pregnancies as the denominator (n = 150043), the incidence of invasive maternal GBS disease was 0.38 (95% CI, .28–.48) per 1000 pregnancies, as shown in Figure 4. From the 4 studies reporting cases of invasive maternal GBS disease by the number of maternities (n = 1576138), the incidence was 0.17 (95% CI, –.01 to .35), also shown in Figure 4.

**Figure 5.**
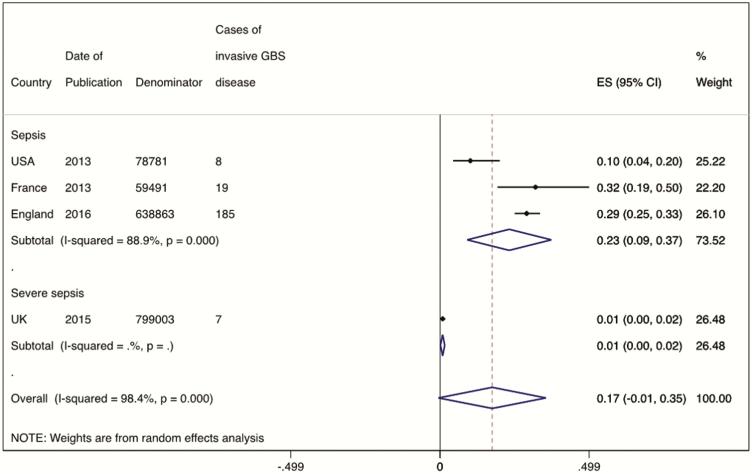
Meta-analysis of the incidence of maternal GBS disease, split by severe sepsis (1 study, n = 799003) or sepsis (3 studies, N = 777135). Abbreviations: CI, confidence interval; ES, effect size; GBS, group B *Streptococcus*.

Most studies included cases of sepsis on the basis of clinical suspicion and positive sterile-site cultures. One study [42] only reported cases of severe maternal sepsis, defined as death related to infection; severe sepsis requiring admission to a high dependency or intensive care unit; or clinical suspicion of sepsis with ≥2 of the symptoms of systemic inflammatory response syndrome (see Supplementary Table 2). By focusing on the most severe cases, this study reported a significantly lower incidence of maternal GBS disease (7 cases in 799003 maternities, incidence 0.01/1000 maternities). A meta-analysis stratified by case definition calculated the incidence of invasive maternal GBS disease from the remaining 3 studies as 0.23 (95% CI, .09–.37) per 1000 maternities (Figure 5).

The studies that reported incidences but could not be included in the meta-analysis are shown in Supplementary Table 3. The incidence appears to have fallen in the United States, from the highest estimate of 2.3 per 1000 deliveries in the late 1970s [44] to 0.12 per 1000 live births in the early 2000s [22]. It should be noted that not all of these studies applied the same definition of a sterile site (for example, some included amniotic fluid, others did not), nor do they use the same denominator and are therefore not strictly comparable either to each other or to the findings of the meta-analysis.

### Timing

The timing of the detection of maternal GBS disease in relation to the course of pregnancy was available for 122 cases from 5 studies [14, 26, 43, 44, 46]. Pooled estimates for the timing of detection show that most cases (66.7% [n = 83]) were detected during labor/delivery (95% CI, 46.6%–86.8%) or postpartum (32.5% [95% CI, 12.1%–52.9%]; n = 37) (see Supplementary Figures 1–3).

### Maternal Outcomes

The overall case fatality risk for pregnant or postpartum women experiencing invasive GBS disease was 0.20% (95% CI, –.40 to .80; 11 studies, 2 deaths, 890 cases) (Supplementary Figure 4). One death occurred in 211 cases (case fatality risk, 0.47% [95% CI, .01–2.61]) [45] and a second, coincidentally, among another 211 cases [22]. The limited data on maternal morbidity are shown in Supplementary Table 4.

### Perinatal Outcomes

From 9 studies where pregnancy outcome was reported [19, 23, 26, 40–42, 44, 46, 47], there were 323 live births, 21 miscarriages, and 14 stillbirths in 357 women with maternal GBS disease. There is some variation in the definition of stillbirth worldwide; not all papers reported their definition, but those that did used 20 or 24 weeks as is common in developed settings. Pooled estimates for pregnancy outcomes were as follows: live births, 93% (95% CI, 88%–98%); miscarriages, 4% (95% CI, 1%–7%); and stillbirths, 3% (95% CI, 1%–5%) (Supplementary Figures 5–7).

**Neonatal Mortality and Morbidity in Babies Born to Women With Maternal Group B *Streptococcus* Disease**

In the 7 studies reporting neonatal mortality, there were 4 neonatal deaths in 160 live births. The pooled estimate for the case fatality risk (all cause) for newborns born to women with maternal GBS disease was 2.2% (95% CI, –1.1% to 5.6%) (Supplementary Figure 8). Of the 4 deaths, no information was given on cause of death for 3; the fourth was a death of a neonate with EOGBS.

In the 6 studies reporting EOGBS, there were 24 cases among 213 live births to women with maternal GBS disease. The pooled incidence estimate for EOGBS was 6.09 (95% CI, .69–11.5) per 1000 live births in women with maternal GBS disease (Supplementary Figure 9). In a case-control study, the infants of mothers with maternal GBS disease had increased odds of either being born prematurely (odds ratio [OR], 6.00 [95% CI, 2.45–14.7] before 37 weeks’ gestation and 13.4 [95% CI, 3.11–57.3] before 32 weeks) or developing sepsis (causative organisms not specified) themselves (OR, 32.7 [95% CI, 8.99–119.0]) [42].

### Colonization

Thirteen neonates who were born to women with maternal GBS disease were colonized among 29 who were tested (44.8%). No information was given on serotypes.

### Serotypes

**Figure 6.**
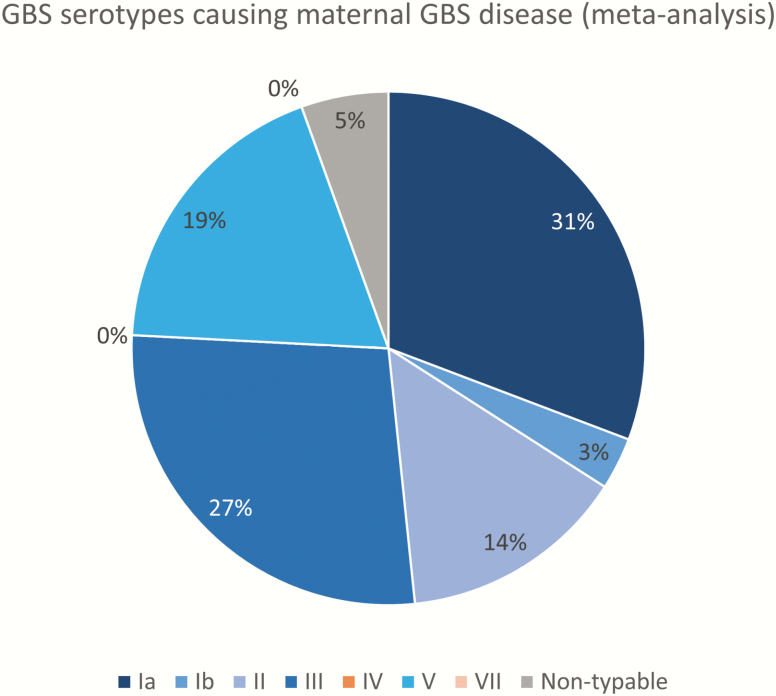
Group B *Streptococcus* (GBS) serotypes causing maternal GBS disease (5 studies, N = 310). Serotypes included in a pentavalent vaccine are shown in blue shades.

Three hundred ten cases were serotyped. The distribution of capsular serotypes causing maternal GBS disease is shown in Figure 6. Serotype Ia was the most common (31%), followed by III (27%), V (19%), Ib (14%), and II (5%).

## DISCUSSION

Our review is the first assessing invasive maternal GBS disease, and we found an incidence of 0.38 (95% CI, .28–.48) per 1000 pregnancies (1 study; 150043 pregnancies) and 0.23 (95% CI, .09–.37) per 1000 maternities in high-income contexts, excluding the study focused solely on severe sepsis. This maternal incidence is lower than the incidence of neonatal GBS disease (0.42 [95% CI, .30–.54]) in developed countries (see Madrid et al in this supplement [33]), but it is likely to be an underestimate due to underreporting and/or low case ascertainment.

While the risk of mortality and morbidity for women with maternal GBS disease appears low in the developed region (case fatality risk, 0.19% [95% CI, –0.25% to 0.62%]), the same cannot be said for the fetus or neonate. Where pregnancy outcomes were known, around 7% of pregnancies ended in miscarriage (4% [95% CI, 1%–7%]) or stillbirth (3% [95% CI, 1%–5%]), and 2.22% (95% CI, –1.11% to 5.55%) of the babies born alive died in the first month of life. These may be underestimates, as some studies did not include the antenatal period (therefore omitting miscarriages and a high proportion of stillbirths) and not all studies followed women up long enough to fully assess pregnancy outcomes. There is a significantly increased risk of EOGBS (6.09 cases [95% CI, .69–11.5] per 1000 live births in women with maternal GBS disease) compared to the background incidence of EOGBS (0.42/1000 live births) [33], and increased odds of preterm birth or sepsis in general [42]. In one study, maternal GBS disease was associated with pregnancy loss or EOGBS in 28% of cases [47]. The risk to the fetus and newborn is likely to be higher in low- and middle-income contexts.

The small number of articles and limited geographies covered, particularly those with pregnancies as a denominator, limit the analysis. However, all data in the studies included in the meta-analysis of the incidence of maternal GBS disease were collected after the year 2000, when the data may be more likely to be comparable. Despite this, there was significant heterogeneity between studies (*I*^2^ = 88.9%). Other important factors for both study heterogeneity and case ascertainment are the case definition employed, the demographic profile of the women included in the study, variations in sampling strategy for the collection of blood cultures in febrile pregnant women and in other clinical practices such as instrumental deliveries and cesarean section rates, the duration of the inclusion period (ie, the length of the antenatal to postpartum period studied), laboratory culture methods used and sensitivity of detection, and use of intrapartum antibiotic prophylaxis and/or antenatal and postnatal antibiotic use (which was poorly reported in these studies), which will reduce detection of GBS [26].

The influence of the case definition used can be seen when comparing the study focusing on severe maternal sepsis with strictly applied clinical criteria, which found the lowest incidence of maternal GBS disease (0.01/1000 maternities) [42], to studies using microbiological results with less stringent clinical criteria, which reported higher incidence risks. For example, GBS bacteremia in febrile pregnant women had a reported incidence of 0.3 per 1000 births [47]. The latter may overestimate cases, as transient bacteremia can occur in the absence of overt clinical sepsis, though in the presence of fever. These inconsistencies in studies purporting to investigate the same issue arise from a lack of consensus on maternal sepsis case definitions [48].

There are further issues with case ascertainment. First, clinical signs of sepsis may be obscured to some extent by the physiological changes in pregnancy [43]. For example, leukocytosis, a sign of sepsis (see Supplementary Table 2), is a normal physiological change in pregnancy; in 1 study only 38% of women with pre- or intrapartum bacteremia had a white cell count outside the normal reference range for pregnancy [43]. Second, many studies looked only at cases of bacteremia, and not at cultures from other sterile sites, underestimating the burden of GBS disease. Third, some febrile pregnant or postpartum women might not have had blood samples taken, reducing case ascertainment. Finally, in up to half of cases of severe sepsis the infection is polymicrobial—that is, a single causative organism cannot be identified from a sterile culture site [49], again leading to an underestimate of the number of cases of maternal GBS disease.

The inclusion period considered by the study could also affect the incidence estimate. A longer postpartum inclusion period could increase case ascertainment, although this may be limited by the fact that most of the postpartum cases occurred within the first 48 hours after delivery, meaning an extended inclusion period may not necessarily detect many more cases. Some studies did not include the antenatal period, or only included the 7 days prior to delivery, which may underestimate cases and lead to an underestimate of the impact of GBS disease on miscarriages and following induced abortion, particularly unsafe abortion. It was not possible to conduct a sensitivity analysis to investigate the effect of the inclusion period as, within the 5 studies included in the meta-analyses, 4 different inclusion periods were used. The categorization of the timing of disease presents a risk of misclassification. For example, a case detected during labor might actually have been an antenatal case where the infection precipitated labor. In the only study where gestational age was reported, 6 cases of maternal GBS disease were identified during labor and 4 of these were premature labor (before 37 weeks), suggesting that these could have been antenatal infections [44].

The complete burden of maternal GBS disease is higher than that estimated here, as there are many other infections caused by GBS. This includes chorioamnionitis (intra-amniotic infection) and postpartum endometritis (PPE), both important contributors to maternal, fetal, and neonatal mortality and morbidity. A study in the United States found that GBS was the most common cause of endometritis or chorioamnionitis [19]. Chorioamnionitis (infection of the intrauterine environment and fetal membranes) affects 1%–4% of pregnancies in developed countries; the incidence in low- and middle-income contexts is not known [50]. In one study, GBS was recovered from amniotic fluid from 15% of women with chorioamnionitis [51]. PPE occurs in around 5% of all vaginal births and 10% of cesarean deliveries in developed countries [52]. Most cases of PPE are not evaluated using sterile site cultures because endometritis is treated empirically [25]. Nonetheless, GBS is an important cause of PPE; a systematic review of 25 studies reported the recovery of GBS from the endometrium in 305 of 3026 (10%) women with PPE [53]. The incidence of PPE, and the contribution of GBS to this, in low- and middle-income contexts is unknown. It is likely to be higher, with fewer deliveries using antisepsis measures and reduced access to antibiotic prophylaxis [54]; however, unpublished data from an ongoing study in Pakistan found that 11 of 468 (2.4%) endometrial samples taken for suspected PPE were positive for GBS (Shakoor et al, for the ANISA-Postpartum Sepsis Study Group, personal communication, 2017). While data from low- and middle-income contexts are limited, the data from developed countries suggest that GBS is an important contributor to total maternal infectious morbidity and perinatal and neonatal mortality and morbidity, even in the absence of systemic infection and sepsis.

We noted an apparent fall in the incidence of maternal GBS disease in the United States, the only country with serial data, from the 1970s to the early 2000s with a possible subsequent plateau (Supplementary Table 3). This is compatible with the decline in the incidence of sepsis noted in developed countries over this time period, recent increases in incidence notwithstanding. It is also in keeping with other findings of a fall in incidence of GBS disease in the United States, which may be due to the introduction of universal screening and increased use of intrapartum antibiotic prophylaxis (IAP) during this time [45]. The fact that screening and IAP cannot prevent all maternal or neonatal disease further supports the case for vaccination prior to labor.

The serotype distribution of maternal GBS disease is, unsurprisingly, similar to that seen in maternal colonization [21] and EOGBS [33]. In all 3 population groups, Ia, III, and V are the most common serotypes causing disease, though there is more serotype III in neonatal disease [33]. Given this similarity, current vaccine candidates are likely to be effective in preventing some maternal GBS disease.

## CONCLUSIONS

This first review of maternal GBS disease suggests that the incidence in developed countries is lower than, but comparable to, neonatal disease in developed countries, and that associated maternal mortality and morbidity are uncommon sequelae. In contrast, the risk for the neonate, in terms of mortality and morbidity, is increased. Given that most maternal cases were peri- or postpartum, maternal vaccination in the late second or early third trimester is likely to be effective at preventing GBS disease in the mother as well as the infant.

The absence of studies from low- and middle-income contexts means these findings cannot be generalized. The incidence of 0.23 (95% CI, .09–.37) per 1000 maternities should be seen as a “minimum estimate” of maternal GBS disease, given the likely higher incidence in the rest of the world, and the fact that we have only considered the more severe, systemic cases and not all infections caused by GBS during pregnancy and the postpartum period.

There is a need for high-quality research into the etiology of maternal sepsis worldwide, but especially in low- and middle-income contexts. This need is credited in the new Global Maternal and Neonatal Sepsis Initiative. This commenced in 2015 under the leadership of the World Health Organization and Jhpiego (an international, nonprofit health organization affiliated with The Johns Hopkins University). The Initiative notes the importance of infection in maternal and newborn morbidity and mortality; the goals include research on the burden and causes, prevention, and management, and information/advocacy [55]. Community-based mother and newborn surveillance systems with identification, appropriate biosampling, and management, such as those implemented in South Asia, can shed further light on the microbiology, along with the epidemiology of maternal infection, including GBS [56].

However, as 80% of the world’s births are now facility based, a routine approach to surveillance is becoming more feasible. National surveillance systems for maternal sepsis would ensure that more attention is paid to pregnant and postpartum women with a fever. Systematically sampling with appropriate laboratory investigation should be done if women have a fever, whether antenatally, during labor, or after birth. Women and their babies should be followed up to assess clinical outcomes. To facilitate international comparisons regarding GBS, agreement on the parameters used to determine cases of maternal GBS disease and the denominator is required. Because sepsis may occur at any stage of pregnancy, and may influence the outcome of pregnancy, the recommended denominator is all pregnant women. Such studies are needed globally, but especially in developing countries to fill this data gap. These data are required to assess the burden of GBS disease and to determine the clinical and cost effectiveness of antenatal screening, treatment, and vaccination strategies to inform policy decisions (Table 2).

**Table 2.** Key Findings and Implications

What’s new about this? • This is the first systematic review and meta-analysis to estimate the incidence of maternal GBS disease worldwide and to describe the causative serotypes and outcomes of disease.
What was the main finding? • The incidence of maternal GBS disease in the developed region is lower than, but comparable to, neonatal GBS disease in the developed region at approximately 0.38 (95% CI, .28–.48) per 1000 pregnancies and 0.23 (95% CI, .09–.37) per 1000 maternities. The serotype distribution of maternal GBS disease is similar to that seen in maternal colonization and early-onset neonatal GBS disease, with serotypes Ia, III, and V most common. The risk of maternal mortality or morbidity is low; however, the risk for the neonate, in terms of mortality and morbidity, is increased.
How can the data be improved? • The incidence of maternal GBS disease in low- and middle-income contexts is an important data gap. To improve the availability and comparability of data, standardized surveillance and reporting systems are required. Agreement is needed on the parameters used to define maternal GBS disease, pregnancies should be used as the denominator, and appropriate follow-up should be conducted to determine the outcome of the pregnancy.
What does it mean for policy and programs? • As most maternal GBS disease is peripartum or postpartum, maternal vaccination in the late second or early third trimester is likely to be effective at preventing GBS disease in the mother as well as the infant. Maternal GBS vaccination would be expected to be more effective than IAP in preventing maternal postpartum GBS sepsis; a maternal vaccine study should measure this outcome and could also contribute to improved measurement of burden as a “vaccine probe” approach.

Abbreviations: CI, confidence interval; GBS, group B *Streptococcus*; IAP, intrapartum antibiotic prophylaxis.

## Supplementary Data

Supplementary materials are available at *Clinical Infectious Diseases* online. Consisting of data provided by the authors to benefit the reader, the posted materials are not copyedited and are the sole responsibility of the authors, so questions or comments should be addressed to the corresponding author.

## Supplementary Material

Supplement_Material

## Notes

***Author contributions.*** The concept of the estimates and the technical oversight of the series was led by J. E. L. and A. C. S.; N. H. A. and F. B. J. performed the database searches. N. H. A., F. B. J., and J. H. removed duplicates and screened titles and abstracts for eligibility. J. H., N. H. A., and F. B. J. read the full texts, confirmed suitability for inclusion, and extracted data. Data analyses were undertaken by J. H.; the first draft of the manuscript was written by J. H. and M. G. G.; T. L. provided input data for the analyses. The GBS Estimates Expert Advisory Group (C. J. B., C. C., P. T. H., M. I., K. L. D., S. A. M., C. E. R., S. K. S., S. S., A. S.-t. M., J. V.) provided input to the conceptual process throughout, notably on the disease schema and data inputs. All the authors reviewed and gave input to the manuscript.

***Acknowledgments.*** The authors thank Claudia da Silva for administrative assistance and Alegria Perez for coordinating author signatures.

***Financial support.*** This supplement was supported by a grant to the London School of Hygiene & Tropical Medicine from the Bill & Melinda Gates Foundation (Grant ID: OPP1131158).

***Supplement sponsorship.*** This article appears as part of the supplement “The Burden of Group B Streptococcus Worldwide for Pregnant Women, Stillbirths, and Children,” sponsored by the Bill & Melinda Gates Foundation and coordinated by the London School of Hygiene & Tropical Medicine.

***Potential conflicts of interest.*** Many contributors to this supplement have received funding for their research from foundations, especially the Bill & Melinda Gates Foundation, and several from Wellcome Trust, Medical Research Council UK, the Thrasher Foundation, the Meningitis Research Foundation, and one individual from the US National Institutes of Health. Members of the Expert Advisory Group received reimbursement for travel expenses to attend working meetings related to this series. A. S.-t. M. works for the Bill & Melinda Gates Foundation. T. L. has received grants from Pfizer Inc to support independent epidemiological studies. C. J. B. has served as a member of the Presidential Advisory Committee for Seqirus Inc and of the CureVac Inc Scientific Advisory Committee, as well as undertaken consultancy work for Pfizer Inc. C. C. has received institutional compensation from Novartis for conducting GBS studies. P. T. H. has been a consultant to Novartis and Pfizer on GBS vaccines but received no funding for these activities. M. I. has undertaken sponsored research from Pfizer on pneumococcal disease in adults and from Belpharma Eumedica (Belgium) on temocillin antimicrobial susceptibility in Enterobacteriaceae. K. L. D. has received funding by the Bill & Melinda Gates Foundation to work on research on GBS serocorrelates of protection to inform vaccine trials, and travel expenses from Pfizer to attend a meeting on an investigator-led project on GBS. S. A. M. has collaborated on GBS grants funded by GlaxoSmithKline and by Pfizer and received personal fees for being member of its advisory committee; he has also collaborated on a GBS grant funded by Minervax. All other authors report no potential conflicts of interest. All authors have submitted the ICMJE Form for Disclosure of Potential Conflicts of Interest. Conflicts that the editors consider relevant to the content of the manuscript have been disclosed.
