# Supplementary material for: Maternal Disease With Group B Streptococcus and Serotype Distribution Worldwide: Systematic Review and Meta-analyses
Source: Clin Infect Dis. 2017 Nov 6;65(Suppl 2):S112–24. doi: 10.1093/cid/cix660 (PMC5850000; doi:10.1093/cid/cix660)
Supplement: Supplement_Material [file cix660_suppl_supplement_material.pdf]

The burden of Group B *Streptococcus* worldwide for pregnant women, stillbirths and children

Paper 3: Maternal disease with Group B *Streptococcus* and serotype distribution worldwide: systematic review and meta-analyses

Supplementary information

Contents

Systematic estimates of the global burden of Group B *Streptococcus* in pregnant women, stillbirths and infants 1

Paper 3: Maternal disease associated with Group B *Streptococcus* and serotype distribution worldwide: systematic review and meta-analyses 1

Supplementary information 1

Supplementary Table S1: Search terms 3

Supplementary Table S2: Systemic Inflammatory Response Syndrome (SIRS) definition - adapted from Surviving Sepsis Campaign 4

Supplementary Table S3: Other incidence estimates of maternal GBS disease 5

Supplementary Table S4: Papers with data on maternal morbidity 6

Supplementary Figure S1: Proportion of maternal GBS disease occurring antenatally 7

Supplementary Figure S2: Proportion of maternal GBS disease occurring during delivery 8

Supplementary Figure S3: Proportion of maternal GBS disease occurring postnatally (up to 42 days) 9

Supplementary Figure S4: Case fatality risk for maternal GBS disease 10

Supplementary Figure S5: Proportion of maternal GBS disease ending in live births 11

Supplementary Figure S6: Proportion of maternal GBS disease ending in miscarriage 12

Supplementary Figure S7: Proportion of maternal GBS disease ending in stillbirth 13

Supplementary Figure S8: Case fatality risk for neonates born to women with maternal GBS disease 14

Supplementary Figure S9: Rate of early onset neonatal GBS disease per 1000 live births to women with maternal GBS disease 15

Supplementary Table S1: Search terms

Maternal OR Mother OR Puerperal OR Parturient OR Antepartum OR Intrapartum OR Peripartum OR Postpartum OR Pregnan$ OR Pregnancy [MeSH Terms]
AND Sepsis OR Septic OR Bacteraemia OR Bacteremia OR Sepsis [MeSH Terms]
AND (“Streptococcus” [All Fields] OR “Streptococcal” [All Fields] OR “Streptococci” [All Fields]) AND ((“Group” AND “B”) or “Agalactiae”) OR “Streptococcus Agalactiae” [MeSH Terms]
**Limit:** humans

Supplementary Table S2: Systemic Inflammatory Response Syndrome (SIRS) definition - adapted from Surviving Sepsis Campaign

**SIRS – Two or more of the following:**
Temperature >38◦C or <36◦C
Heart rate >90 beats per minute
Respiratory rate > 20/min
White cell count >12,000µl-1 or <4,000µl-1

Supplementary Table S3: Other incidence estimates of maternal GBS disease

**Study**	**Pass**	**Schwartz**	**Schrag**		**Tyrell**	**Zalzenik**	**Phares**	**Deutscher**
**Year**	**1982**	**1991**	**1993**	**1998**	**2000**	**2000**	**2008**	**2011**
**Country**	**USA**	**USA**	**USA**		**Canada**	**USA**	**USA**	**USA**
Rate	2.3 and 1.4 per 1,000 deliveries	0.22/1000 live births[1]	0.29/1000 live births[2]	0.23/1000 live births[2]	0.41/1000 live and stillbirths[3]	0.3/1000 deliveries[4]	0.12 (0.11-0.14)/1000 live births[5]	Pregnancy: 0.02/1000 women years Postpartum: 0.4/1000 women years[6]

[1]14 cases: 9 bacteremic, 5 endometrial or placental cultures. [2] Normally sterile site; amniotic fluid, placenta or urine alone excluded. [3] 15 cases: 11 bacteremic, 3 amnion/chorioamnion or amniotic fluid, 1 cord blood culture. [4] Sterile body site excluding urine. 54 cases, 52 bacteremic, 2 amniotic fluid from amniocentesis with intact membranes. [5] Normally sterile site, 409 cases, 211 bacteremic, 187 placenta, amniotic fluid or conceptus, 8 from peritoneal fluid, 3 other sterile site. NOTE GBS isolated from a sterile site in a stillborn baby, or from placenta, amniotic fluid or conceptus in context of fetal death were classified as maternal cases. [6] Normally sterile site; amniotic fluid, placenta or urine alone excluded. 99 cases.

Supplementary Table S4: Papers with data on maternal morbidity

**Kalin (n=7)**	**Knowles (n=348)**	**Surgers (n=19)**	**Phares (n=409)**	**Deutscher (n=99)**
Pulmonary oedema 1; Coagulopathy 1; Postpartum Haemorrhage 3; Retroperitoneal haematoma 1; Thrombocytopaenia 1; Bilateral iliopsoas abscess 1; Necrotising fasciitis 1	HDU Admission 4 for GBS of 20 admissions	No ITU admission, no septic shock; no other morbidity data given.	Endocarditis 1; Pneumonia 8;	"High case fatality rate syndrome" 19 (pregnant) 24 (postpartum)

Supplementary Figure S1: Proportion of maternal GBS disease occurring antenatally

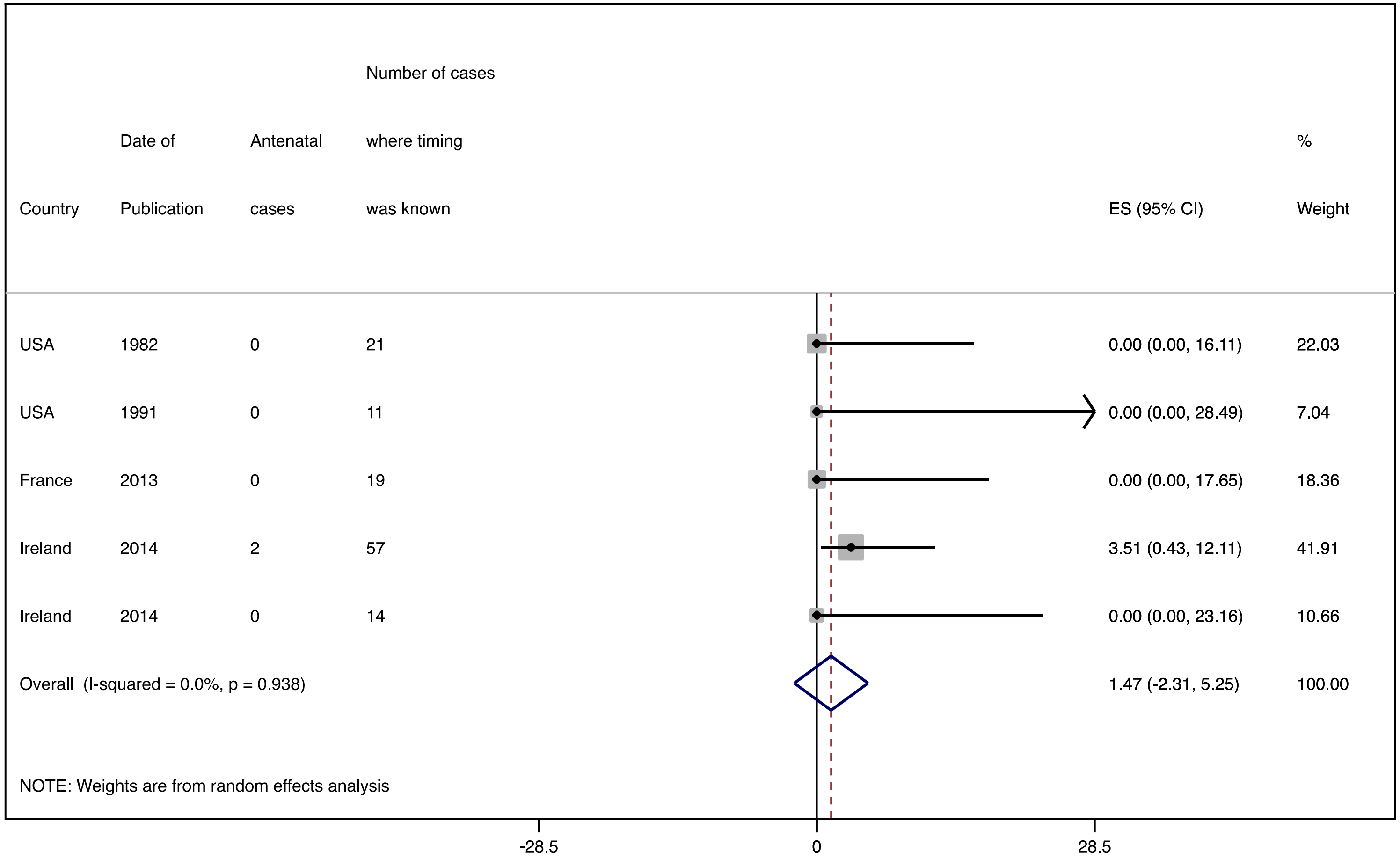

Supplementary Figure S2: Proportion of maternal GBS disease occurring during delivery

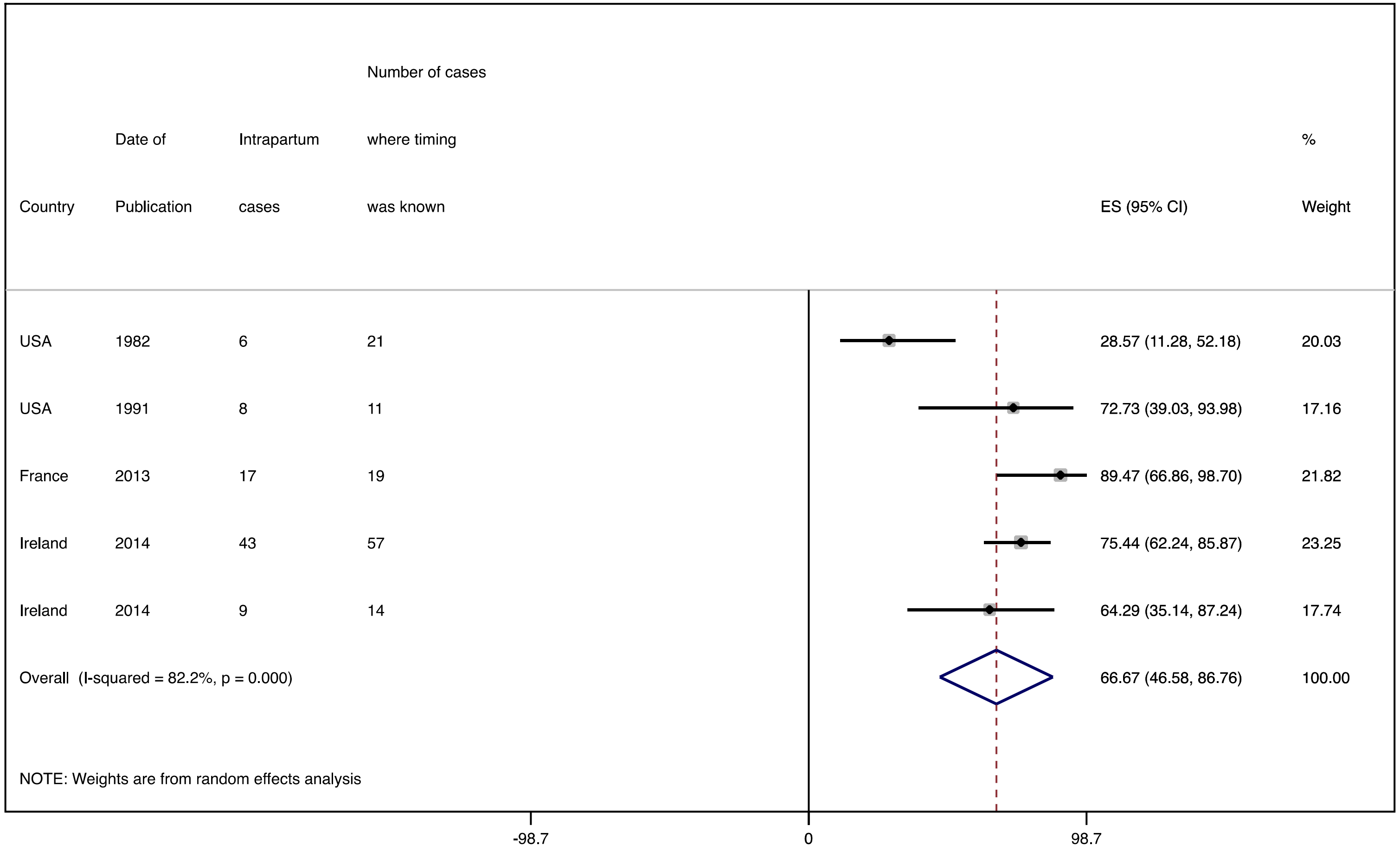

Supplementary Figure S3: Proportion of maternal GBS disease occurring postnatally (up to 42 days)

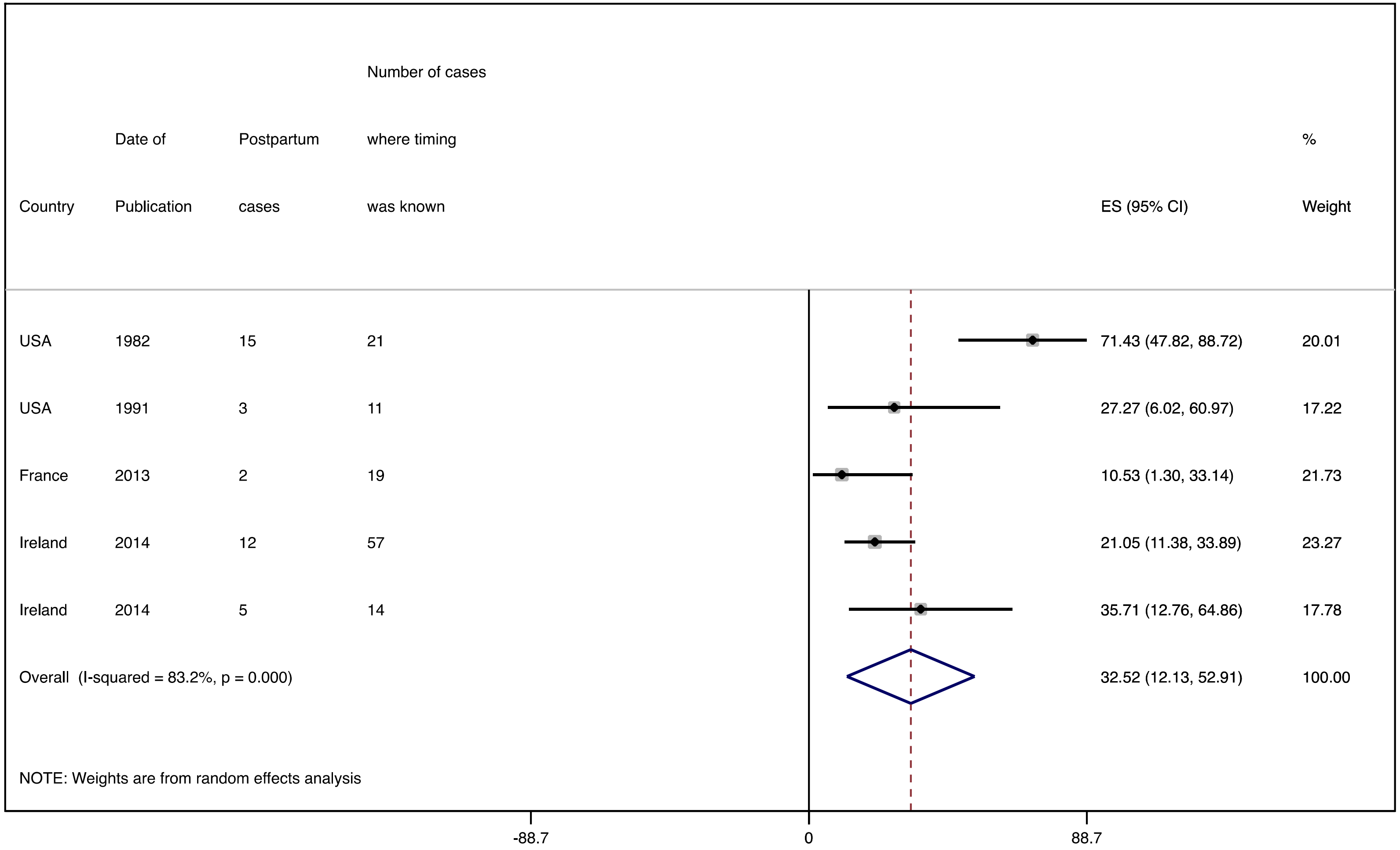

Supplementary Figure S4: Case fatality risk for maternal GBS disease

Country	Date of Publication	Maternal deaths	Cases of invasive GBS disease	ES (95% CI)	% Weight
USA	1982	0	21	0.00 (0.00, 16.11)	0.56
USA	1985	0	4	0.00 (0.00, 60.24)	0.04
Canada	2000	0	15	0.00 (0.00, 21.80)	0.31
USA	2000	0	54	0.00 (0.00, 6.60)	3.34
USA	2000	1	211	0.47 (0.01, 2.61)	21.53
USA	2008	1	211	0.47 (0.01, 2.61)	21.53
USA	2011	0	99	0.00 (0.00, 3.66)	10.88
France	2013	0	19	0.00 (0.00, 17.65)	0.47
Ireland	2014	0	57	0.00 (0.00, 6.27)	3.71
Ireland	2014	0	14	0.00 (0.00, 23.16)	0.27
England	2016	0	185	0.00 (0.00, 1.97)	37.35
Overall (I-squared = 0.0%, p = 1.000)				0.20 (-0.40, 0.81)	100.00

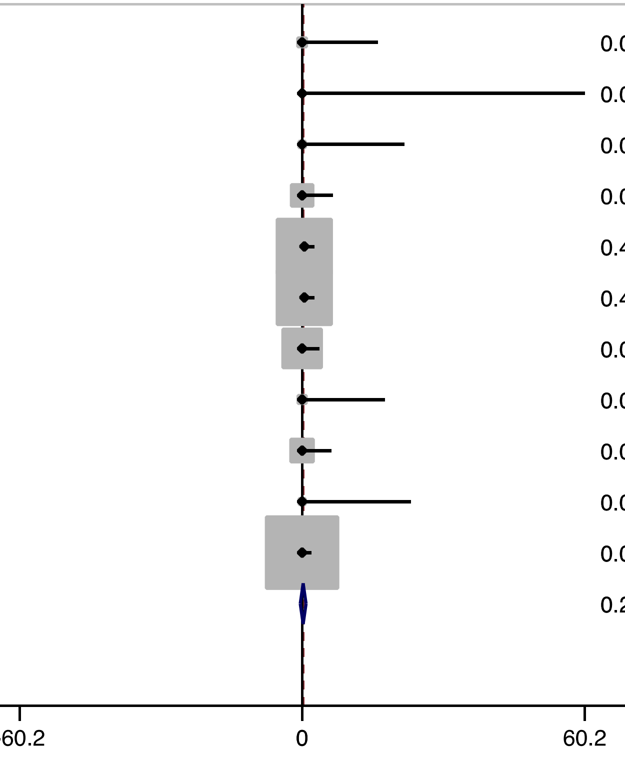

NOTE: Weights are from random effects analysis

Supplementary Figure S5: Proportion of maternal GBS disease ending in live births

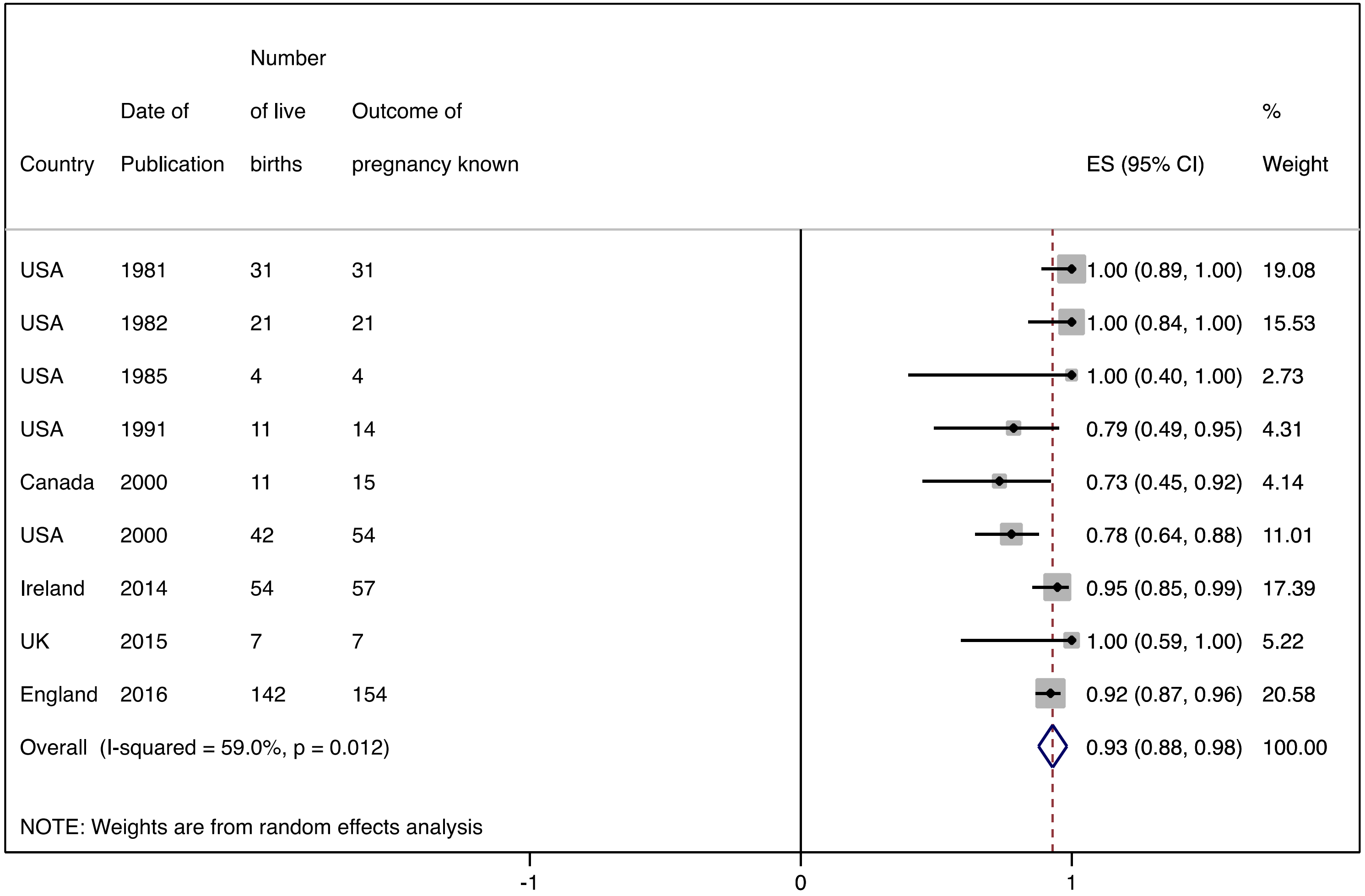

Supplementary Figure S6: Proportion of maternal GBS disease ending in miscarriage

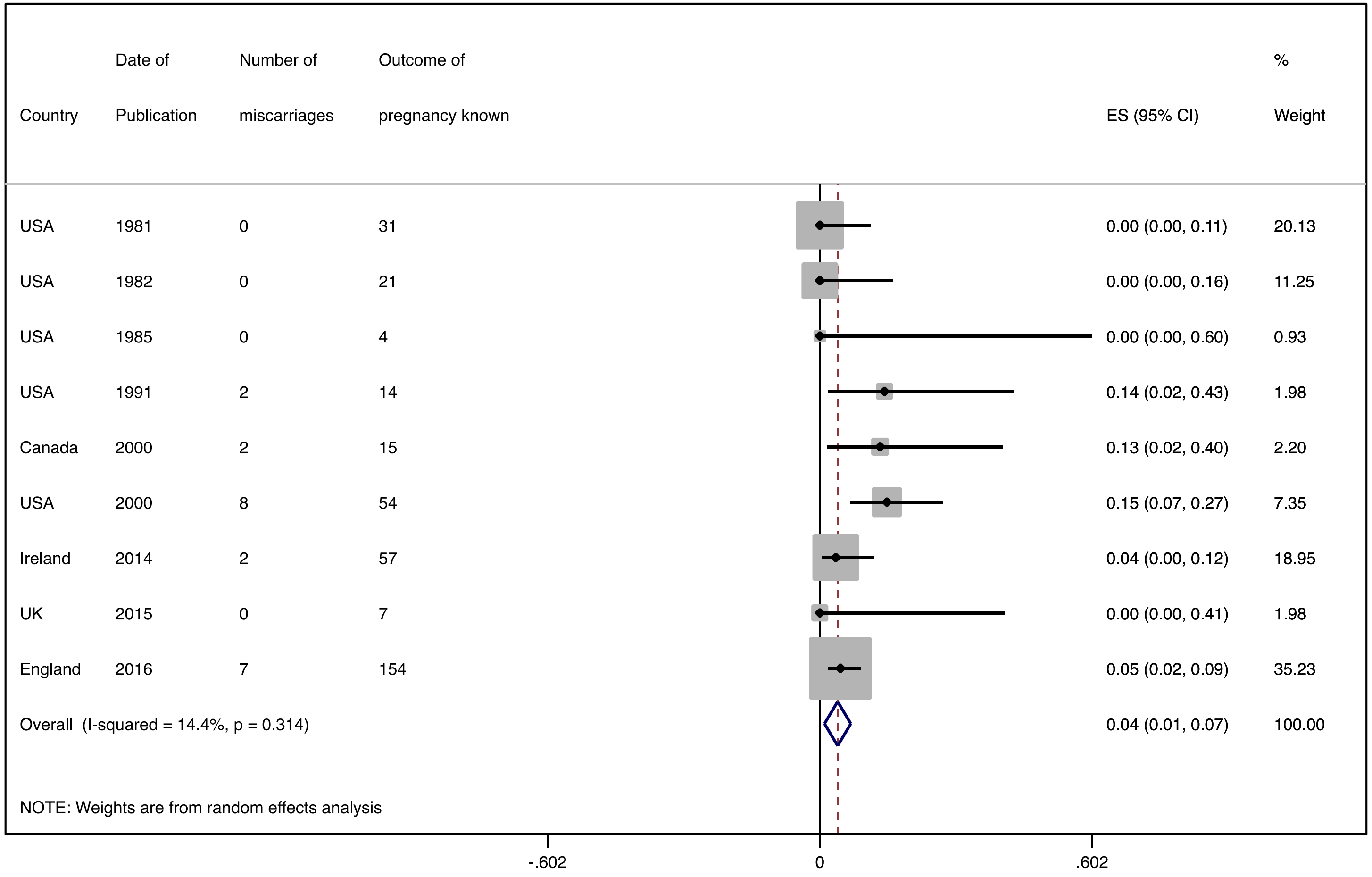

Supplementary Figure S7: Proportion of maternal GBS disease ending in stillbirth

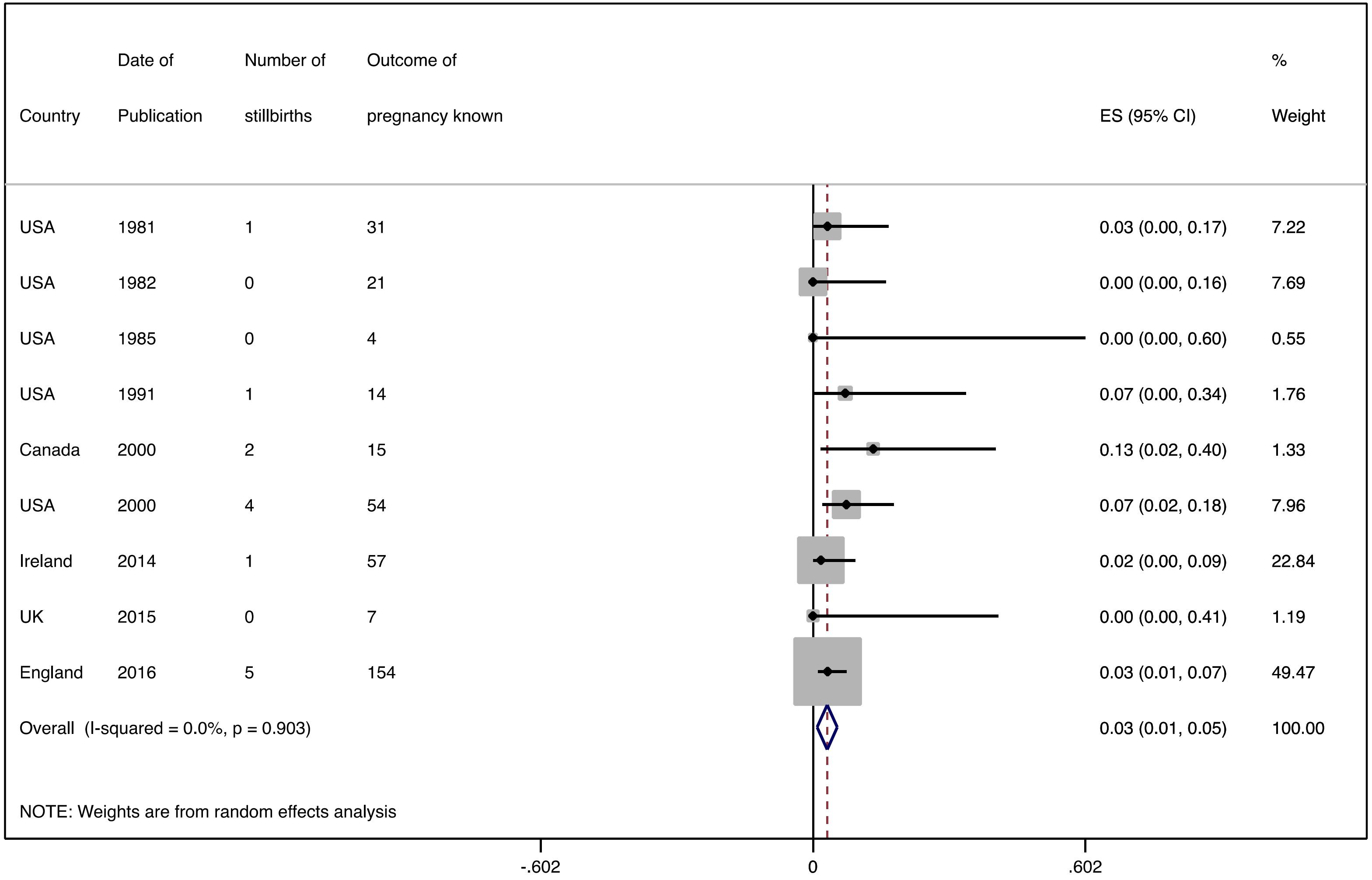

Supplementary Figure S8: Case fatality risk for neonates born to women with maternal GBS disease

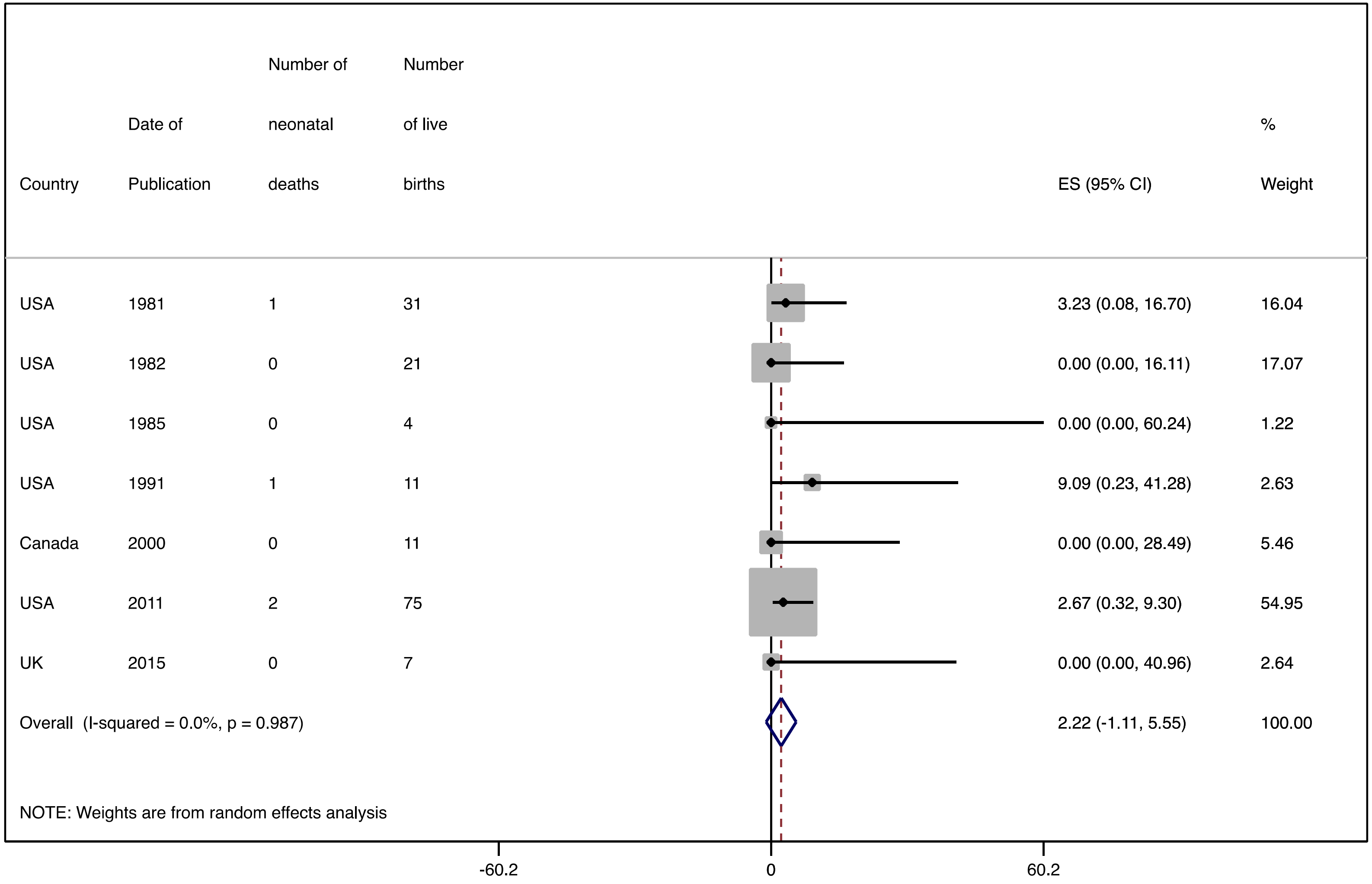

Supplementary Figure S9: Rate of early onset neonatal GBS disease per 1000 live births to women with maternal GBS disease

Country	Date of Publication	Cases of EOGBS	Number of live births	ES (95% CI)	% Weight
USA	1981	2	31	6.45 (0.79, 21.42)	27.33
USA	1982	2	21	9.52 (1.17, 30.38)	13.64
USA	1985	1	4	25.00 (0.63, 80.59)	1.82
Canada	2000	0	11	0.00 (0.00, 28.49)	14.33
USA	2000	3	42	7.14 (1.50, 19.48)	35.96
UK	2015	0	7	0.00 (0.00, 40.96)	6.93
Overall (I-squared = 0.0%, p = 0.825)				6.09 (0.69, 11.48)	100.00

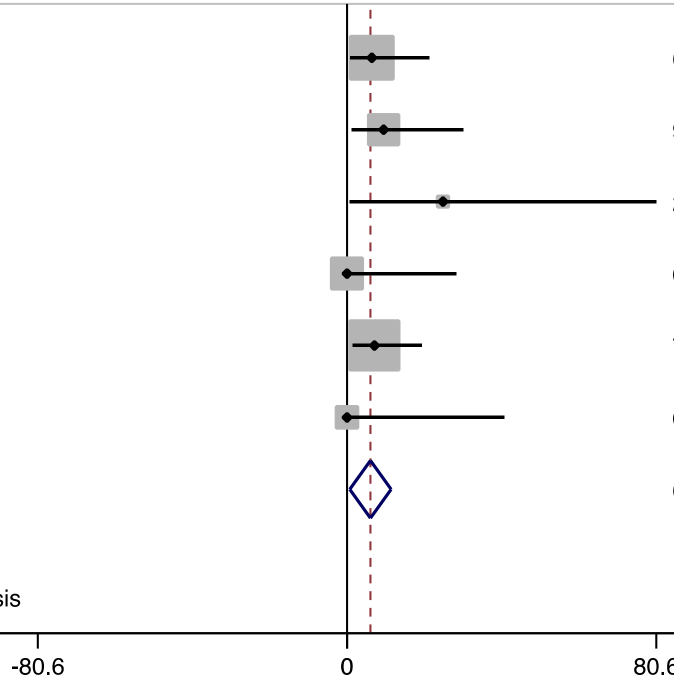

NOTE: Weights are from random effects analysis
